# Evidence of an increased incidence of myocardial inflammation associated with reduced ventricular function in clinically suspected idiopathic dilated cardiomyopathy - a cardiovascular magnetic resonance study

**DOI:** 10.1186/1532-429X-13-S1-P266

**Published:** 2011-02-02

**Authors:** Oliver Strohm, Myra S Cocker, Tak S Fung, Saleh Kary, Helene Childs, Matthias G Friedrich

**Affiliations:** 1 Stephenson Cardiovascular Magnetic Resonance Centre, Libin Cardiovascular Institute of Alberta, Departments of Cardiac Sciences and Radiology, University of Calgary, Calgary, AB, Canada; 2 Department of Information Technologies, University of Calgary, Calgary, AB, Canada

## Background

Dilated cardiomyopathy (DCM) occurring due to an unknown etiology or genetic predisposition is termed as idiopathic dilated cardiomyopathy (iDCM), although iDCM may also result from viral exposure. However, the incidence of myocardial inflammation and its relation to left ventricular (LV) function in iDCM remains unknown.

Cardiovascular magnetic resonance (CMR) imaging allows for the visualization of myocardial inflammation using early Gadolinium enhancement (EGE). We applied EGE imaging in the setting of clinically suspected iDCM to determine both the incidence and relation of myocardial inflammation to LV function.

## Methods

26 patients (17 males, age 44±14 years old) were referred to us for the assessment of iDCM following a clinical suspicion of iDCM, based upon the following criteria: ejection fraction below 45%; invasive exclusion of significant coronary artery disease using a cutoff of 50% stenosis; stable clinical course for at least 3-months prior to the CMR study; exclusion of myocarditis within the past 12 months; as well as exclusion of comorbidities which may otherwise account for patient presentation including valvular heart disease.

Standard CMR imaging procedures for the assessment of LV function and EGE were utilized. EGE images were acquired before and early after (over 4 minutes) Gd-DTPA 0.1ml/kgBW contrast injection using T1-weighted images.

LV function was assessed by manually tracing endo- and epicardial contours. Myocardial signal intensity was normalized to skeletal muscle, generating a ratio that had to be greater than or equal to 4 to be considered positive for EGE.

## Results

**Figure 1:**
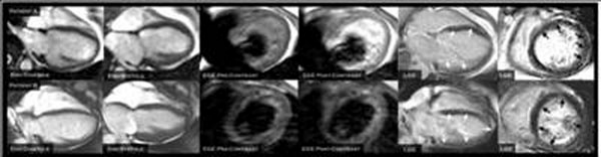
Significant ventricular dilatation and severely reduced global contractile in patient A (LV end-diastolic volume indexed-to-height 224 ml, end systolic volume indexed-to-height 166 ml, ejection fraction 25.8%) who has visibly increased early enhancement (EE ratio 11.2), compared to patient B with lack of early enhancement (EE ratio 2.0) and moderately ventricular function (LV end-diastolic volume indexed-to-height 162 ml, end-systolic volume indexed-to-height 87 ml, ejection fraction 42.5%). Note that both patients have evidence of myocardial fibrosis (arrows).

Eighteen patients (69%) presented with EGE and a significantly increased EGE ratio (7.4±3.6 vs. 2.9±0.7, p=0.002). The ratio of EGE correlated with LVEDVI (r=0.530, p=0.005) and LVESVI (r=0.596, p=0.001), LVSVI (r=-0.437, p=0.260), and LVEF (r=-0.633, p=0.001). Patients with elevated EGE had significantly dilated left ventricles and globally reduced ventricular function: LVEDVI (167.1±47.8 ml/m vs. 111.9±28.6 ml/m, p=0.006) and LVESVI (130.9±48.0 ml/m vs. 57.3±17.3 ml/m, p<0.001) were increased, while LVSVI (36.2±14.2 ml/m vs. 55.6±13.6 ml/m, p=0.005) and LVEF (23.3±10.6% vs. 49.1±5.49%, p<0.001) were reduced (Figure 1).

## Discussion

Using CMR-based EGE as a surrogate marker of myocardial inflammation, we provide first evidence for a high incidence of inflammation in patients with clinically suspected iDCM. The extent of myocardial enhancement was directly related to reduced ventricular function. CMR-based assessment of myocardial inflammation may be utilized as a biomarker for patient prognosis and guide medical therapy to target those patients in whom there is active myocardial inflammation.

